# Textiles Functionalized with Copper Oxides: A Sustainable Option for Prevention of COVID-19

**DOI:** 10.3390/polym14153066

**Published:** 2022-07-29

**Authors:** Luz Esmeralda Román, Cleny Villalva, Carmen Uribe, Francisco Paraguay-Delgado, José Sousa, Johnny Vigo, Concepción Mercedes Vera, Mónica Marcela Gómez, José Luis Solís

**Affiliations:** 1 Faculty of Science, Universidad Nacional de Ingeniería, Av. Túpac Amaru 210, Lima 15333, Peru; luz.esmeralda.roman@uni.pe (L.E.R.); mgomez@uni.edu.pe (M.M.G.); 2 Facultad de Ingeniería Química y Textil, Universidad Nacional de Ingeniería, Av. Túpac Amaru 210, Lima 15333, Peru; cvillalvac@uni.pe (C.V.); curibe@uni.edu.pe (C.U.); 3 Centro de Investigación en Materiales Avanzados SC. (CIMAV), Chihuahua CP 31136, Mexico; francisco.paraguay@cimav.edu.mx; 4 Tejidos San Jacinto S.A., Av. Colectora Industrial 162, Lima 15011, Peru; jose.sousa@sanjacinto.com.pe (J.S.); johnny.vigo@sanjacinto.com.pe (J.V.); concepcion.vera@sanjacinto.com.pe (C.M.V.)

**Keywords:** textile, functional, copper oxides, sustainable, COVID-19, antimicrobial, industrial scale

## Abstract

COVID-19 is caused by severe acute respiratory syndrome coronavirus 2 (SARS-CoV-2), and healthcare-associated infections (HAIs) represent severe problems in health centers and public areas. Polyester/cotton (PES/CO) blend fabrics have been functionalized with copper oxides on an industrial scale. For functionalization, the impregnation dyeing technique was applied. The functionalized samples were tested virologically against SARS-CoV-2 and human coronavirus (229E) according to ISO 18184-2019 and microbiologically against *Escherichia coli* (ATCC 25922) bacteria according to ASTM E2149-2013. The results show that the fabric functionalized with copper oxides inactivated both viruses after 30 min of exposure, presenting excellent virucidal activity against 229E and SARS-CoV-2, respectively. Furthermore, its inactivation efficiency for SARS-CoV-2 was 99.93% and 99.96% in 30 min and 60 min exposure, respectively. The fabric inhibited bacterial growth by more than 99% before and after 10 and 20 washes. In conclusion, 265 m of PES/CO fabric (wide 1.7 m) was functionalized in situ on an industrial scale with copper oxide nanoparticles. The functionalized fabric presented virucidal and bactericidal properties against SARS-CoV-2 and *Escherichia coli*.

## 1. Introduction

An increase in harmful pollutants caused by anthropogenic activities such as deforestation, transportation, agricultural expansion, industrial processes, increased wastewater, and others, and global climate change, which causes an increase in the average global temperature and changes in rainfall patterns, among other things, pose a significant threat to human health and other forms of life [1,2,3]. Likewise, with urbanization, natural habitats of animals are being encroached on; this activity threatens the ecology and movement of the animal population and creates a great possibility of the exchange of diseases between humans and wildlife [4,5]. An example of how an animal virus jumped species boundaries and was able to infect humans so productively is the severe acute respiratory syndrome coronavirus 2 (SARS-CoV-2), which causes coronavirus disease 2019 (COVID-19); that is, this new virus arose in the human population from an intermediate animal host [6,7]. The COVID-19 disease is highly contagious and has different variants [8,9]. According to Sturdy et al. (2020), there is a significant risk of increased morbidity and mortality rates of the sickest COVID-19 patients due to neglect of hospital-acquired infection control, also known as healthcare-associated infections (HAIs) [10]. These infections are preventable side effects, and they are regarded as signs of high-quality patient care [11,12] Some of the recommendations to avoid contracting and spreading HAIs, mainly COVID-19, are hand hygiene, use of alcohol gel, disinfecting surfaces, not having direct contact with people who contracted the virus, and use of personal protective equipment (PPE) [13,14]. PPE includes gloves, face covering, surgical masks, high-filtration masks, reusable face masks, and protective clothing, among others [13]. Therefore, using reusable materials for manufacturing PPE, such as fabrics with antimicrobial properties functionalized with different metals and/or semiconductor oxides, could prevent and reduce the spread of viruses or other pathogens. Abou Elmaaty et al. (2022) obtained a polyester fabric with antiviral and antibacterial properties. Using the screen-printing technique, they deposited a paste containing selenium nanoparticles, thickener, and binder into the polyester. The fabric obtained inactivated the SARS-CoV-2 and inhibited the growth of *Bacillus cereus*, *Pseudomonas aeruginosa*, *Salmonella typhi*, and *Escherichia coli* bacteria [15]. Kumar and colleagues (2021) coated the surface of cotton cloth with silver nanoparticles (Ag NPs) by photo-depositing using UV irradiation. Before functionalization the cotton cloth, the authors prewashed the cotton cloth several times ultrasonically in a mixture of acetone and distilled water. The washed cloth was immersed in an aqueous solution of 1 mM silver nitrate, which was kept under the UV lamp for 30 min; then, the cloth was dried at 50 °C. The cloth functionalized with Ag NPs presented virucidal properties against the SARS-CoV-2 virus with a 97% efficiency. Furthermore, it exhibited bactericidal and fungicidal properties against *Escherichia coli* and *Aspergillus Niger* [16]. Roy et al. (2020) synthesized and coated cotton fabric with ZnO nanoparticles using the dip coating technique. They found that the fabric with 2 M ZnO had antibacterial activity against *Staphylococcus aureus* and *Escherichia coli* strains and antifungal activity against *Aspergillus Niger* [17]. As can be found in the literature, there are different processes and techniques for functionalizing fabric surfaces, such as sol–gel, cross-linking, plasma surface activation, and others [18], as well as functionalization methods to obtain fabric with antimicrobial properties. The functionalization methods can be ex situ or in situ [19]. The in situ functionalization of fabrics with copper oxides can be through techniques such as sonochemistry, dip coating, dip coating + shaking, exhaustion dyeing, and others [20,21,22]. Cotton is a vegetable fiber that is predominantly made up of cellulose. Cotton cellulose is a linear, highly crystalline polymer and oriented [23]. It is made up of β-1,4-D(+)-glucopyranose building blocks in long cellulose chains linked by 1,4-glucodic bonds [23]. Polyester (PES) are polymers generated through condensation reactions [24]. Polyester fibers are synthetic fibers used in industrial manufacturing [25]. These fibers are composed of at least 85% by weight of an ester of dihydric alcohol and terephthalic acid [26]. Fibers are the basic units of yarns, and yarns are the building block of a woven fabric. Woven fabrics consist of interlacements of yarns (warp and weft) in two perpendicular directions [27]. Fabric testing provides information about the physical and performance properties of fabrics. Physical properties include fabric thickness, width, weight, and the number of yarns per unit fabric area. Performance properties include strength, abrasion resistance, pilling, and colorfastness [28]. Colorfastness is the resistance of a material to change or transfer its color characteristics to adjacent materials due to its exposure to any external agents [29]. Fastnesses are evaluated with grey scales for color change and staining; scales consist of pairs of standard grey chips representing progressive differences in color and are divided into different grades; higher grade 5 means good colorfastness, and grade 1 means poor colorfastness [29].

In the present research was produced a PES/CO fabric with antiviral and antimicrobial properties, functionalized in situ with copper oxides, produced at the industrial scale using the impregnation dyeing method and essential and pre-existing equipment in the wet processing textile industry. This fabric could be used as a manufacturing material for reusable PPE in indirect contact with human skin.

## 2. Materials and Methods

### 2.1. Materials

Technical-grade copper sulfate pentahydrate (CuSO_4_·5H_2_O) and sodium hydroxide (NaOH) were purchased from a local company. The material used to achieve textiles with antiviral and antibacterial properties was a 65/35 polyester/cotton (PES/CO) blend fabric (2/2 twill weave, weight: 210.7 g/m^2^, warp and weft yarn title: 30/1 Ne, wide: 1.7 m) obtained from a local fabric factory. Other products with analytical grades and equipment used in this study are described in the following subsections.

### 2.2. Functionalization Process at Industrial Scale

In situ functionalization of fabrics with copper oxides were conducted at Tejidos San Jacinto S.A. textile factory facilities in Lima, Peru. The method used was impregnation with the pad–dry/pad–dry/pad–thermofix process. Two hundred sixty-five meters of PES/CO fabric were used for industrial production, and 300 L of the total volume of copper sulfate and sodium hydroxide solutions were employed to synthesize copper oxides. Functionalization at the industrial scale consisted of: Step 1: Pad–dry—Cu^2+^ ion impregnation: A 7.7 g/L aqueous solution was prepared from CuSO_4_·5H_2_O in the 700 L padding machine’s vat. The ready-to-dye dry fabric was submerged in the above-mentioned solution and went through the padding machine’s padding rollers at 15 m/min speed. The rollers were previously calibrated at a pressure of 1 bar. Infrared rays were then used to pre-dry the fabric. Step 2: Pad–dry—NaOH impregnation: A 8.0 g/L NaOH solution was made, and the dried fabric from step 1 was soaked in it. The fabric passed through the padding rollers calibrated at a pressure of 1 bar. Then the fabric was pre-dried with infrared rays. Step 3: Pad–thermofix—copper oxides fixation: The fixation of copper oxides in the fabric obtained in step 2 was carried out in a stenter frame machine at the temperature of 202 °C for 90 s. Step 4: After-treatment—removal of copper oxides not fixed to the fabric: The functionalized fabric with copper oxides was rinsed with soft water at 60 °C and 40 °C, neutralized with 0.5 g/L acetic acid solution at 40 °C and rinsed again at 40 °C. Step 5: Drying: The fabrics functionalized with copper oxides after fixing and washing were dried at a temperature of 100 °C. The functionalization mentioned is described in the patent granted to the present research group [30].

### 2.3. Characterization Techniques, Quality Control, and Physical Properties

The chemical environment of the elements on the functionalized fabric surfaces was determined by X-ray photoelectron spectroscopy (XPS) using a Thermo Fisher Scientific ESCAlab 250Xi spectrometer modulated with a monochromatic Al Kα X-ray source at 1486.6 eV. The morphology of the copper oxide was studied by TEM using a JEOL model JEM2200FS transmission electron microscope, operating at 200 kV with a spherical aberration corrector unit for STEM mode. The sample for TEM was extracted from the warp and weft yarns from the functionalized fabric, which was untwisted to obtain fibers. The fibers were immersed in a vial where isopropyl alcohol was added, which was dispersed by ultrasound bath for 10 min, then it was left to stand for 15 min, and then the fibers were grouped and removed from the vial. Two drops of the dispersion were placed on a nickel 400 mesh grid with a lacey carbon support film. Finally, the grid was allowed to dry at 50 °C for 15 min. Other spectroscopy techniques, such as SEM, EDS, FTIR, and XRD, were attempted. However, no signal was detected due to the low quantity of copper oxide on the textile (below 0.1 %wt.).

Quality control was carried out using protocols of international technical standards. The colors can fade or change when the textile is exposed to water, light, rubbing, washing, or perspiration, so the colorfastness of textile is the resistance to change in any of its color characteristics, including the transfer of its colorants to adjacent materials. Colorfastness to laundering was determined according to AATCC 61 Method 2A—2013e (2020) [31], washing a multifiber fabric attached to the functionalized fabric in 150 mL of a solution with 0.15% standard reference detergent without optical brightener (WOB) with 50 stainless steel balls in a 500 mL steel beaker. The beaker was closed and placed inside the laundering machine previously heated at 49 °C and maintained a constant rotation speed of 40 rpm for 45 min. At the end of the washing process, the test specimen was rinsed three times with distilled water at 40 °C for 1 min and dried at 70 °C. Greyscales evaluate the AATCC fastness ratings for bleeding and fading. They consist of five different grey color appearances, and they are compared with tested textile and its prescribed adjacent material.

The colorfastness to perspiration was evaluated with the AATCC 15:2013e method [32]. The acidic perspiration solution of pH 4.3 was obtained by adding 10 g sodium chloride, 1 g lactic acid, 1 g sodium phosphate, dibasic, anhydrous, and 0.25 g L-histidine monohydrochloride. The test specimens were obtained by attaching equal areas (90 mm × 20 mm) of a multifiber fabric with the functionalized fabric and were placed inside Petri dishes, where the previously prepared perspiration solution was added. The test specimens were wholly submerged in the perspiration solution for 30 min at room temperature, then wrung and placed between plexiglass or glass plates in the perspiration tester and introduced into an oven for 6 h at 38 °C. Then, the specimens were removed and dried at room temperature, and the fastness ratings were evaluated.

Colorfastness to crocking was carried out according to AATCC 8:2016e [33]. For this test, the functionalized fabrics with copper oxides were initially conditioned for 4 h at a temperature of 21 °C ± 1 °C and 65 ± 2% relative humidity. Once the conditioning was finished, 50 mm × 130 mm functionalized fabric was placed on the crockmeter’s abrasive cloth and fixed to the base of the same crockmeter with the help of a specimen holder. In this test, two types of crocking fastness were performed: dry and wet. A white test cloth square was placed on the meter finger for a dry crocking test, which projects downward from the weighted sliding arm and is held with the spiral wire clip. Then, the finger was lowered onto the functionalized fabric, and the meter handle began to rotate 10 complete turns at a rate of one turn per second. The white test was removed for subsequent evaluation at the end of this part. For the wet crocking test, a white test cloth square was moistened with distilled water until a pick-up of 65 ± 5% was achieved. The procedure continued following the same steps used in dry crocking. After 10 turns of the meter handle, the white test was removed and allowed to dry in the environment, finally carrying out each one to evaluate the fastness ratings.

Colorfastness to light was determined according to AATCC 16 (opt 3):2020 [34] and consisted of placing a 70 mm × 120 mm sample of the functionalized fabric and the L4 AATCC blue wool lightfastness standard in the frames supplied with the xenon-arc test apparatus. Subsequently, the apparatus was operated at the selected temperature, humidity, and option for 20 h with continuous light. Once the exposure was completed, the samples were removed from the apparatus to evaluate each one the fastness ratings.

The physical properties were evaluated according to the ASTM D3775:17e1 [35], ASTM D1424:09 (2013) e1 [36], and ASTM D5034:09 (2017) [37] standards, which are methods to assess the density of the yarn, tearing strength, and breaking strength of fabrics, respectively. The results were reported in Newtons (N) for each strength test.

### 2.4. Antiviral and Antibacterial Evaluations

The antiviral property was tested following the methodology described in ISO 18184 [38]. Two viral species and two host cell lines were employed. The two viral species were SARS-CoV-2_COV2019 ITALY/INMI1 and Human Corona Virus 229E strain ATCC VR-740. The host cell lines were VERO E6 for the SARS-CoV-2 virus and MRC-5 cells ATCC CCL-171 for the human coronavirus 229E. The viral suspensions were prepared according to the methodology of ISO 18184. The growth medium was drained from flasks containing VERO E6 and MRC-5 cells cultured in a monolayer at 37 °C under 5% CO_2_ for five days. The surface of the cultured cells was washed twice with the maintenance medium prepared from eagle’s minimum essential medium (EMEM). Then, this surface was inoculated with a virus suspension prepared to a concentration of approximately 10^3^ Plaque Forming Units (PFU) per mL or TCID_50_/mL. The flask containing the cells and the virus was placed in a CO_2_ incubator at 34 °C for 1 h. After this period, 20 mL of maintenance medium and 30 µL of trypsin derived from beef pancreas were added to the flask, and this preparation was placed in a CO_2_ incubator at 34 °C for 1 or 3 days to multiply the viruses. The multiplied viruses were centrifuged for 15 min at 4 °C and 1000 g. The supernatant suspension was then taken from the centrifugation tube. Finally, it was verified that the virus concentration of the supernatant suspension taken from the centrifugation tube was found to be greater than 10^7^ PFU/mL or TCID_50_/mL. Subsequently, nine non-functionalized PES/CO fabrics were prepared to be used as control samples, and six specimens from functionalized fabric with copper oxides. The samples with and without functionalization were 20 mm × 20 mm; they were sterilized at 121 °C for 15 min using 0.2 mL of initially prepared virus suspensions, and textile specimens contained in vials were inoculated. Immediately after inoculation, 20 mL of a wash solution was added to the vial containers of 03 control samples to wash out the virus from the specimens. The remaining vials were placed in the CO_2_ incubator at 34 °C for a contact time of 30 and 60 min. After this contact time, 20 mL wash solution was added to wash out the virus from the specimens. Finally, the obtained wash-out virus suspensions were diluted for subsequent measurement, and the reduction rate was calculated by comparing the antiviral functionalized specimen and control specimen by common logarithm.

Antibacterial analysis of non-functionalized and in situ functionalized PES/CO fabric with copper oxides was performed according to the ASTM E2149:2013 standard [39]. In this evaluation, the *Escherichia coli* (ATCC 25922) strain was grown in sterile tryptic soy broth (TSB) for 18 h at 35 °C. After that, the strain was washed with a sterile buffer solution (0.3 mM KH_2_PO_4_) and diluted until an absorbance of 0.28 ± 0.02 at 475 nm was achieved, equivalent to 1.5–3.0 × 10^8^ colony-forming units (CFU) per milliliter. This bacterial suspension was further diluted, and a final concentration of 1.5 × 10^5^ CFU/mL was obtained (working bacterial solution). Afterward, one gram of fabric was placed inside an Erlenmeyer flask containing 50 mL of working dilution of bacterial inoculum. The flask was incubated at 35 °C and continuously shaken at maximum revolution for 1 h. After incubation, the bacterial solution, which was in contact with the fabric sample, was diluted and plated in triplicate on a Petri dish containing tryptic soy agar (TSA). All the Petri dishes were incubated at 35 °C for 24 h. Then the colonies in each Petri dish were counted, and the percentage of bacterial reduction was calculated.

## 3. Results and Discussions

**Figure 1:**
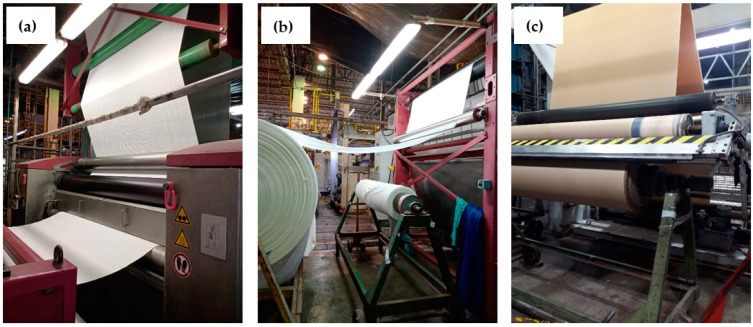
Industrial scaling: (**a**) before, (**b**) during, and (**c**) after the functionalization process of the PES/CO fabric with copper oxides.

The 265 m of PES/CO fabric was functionalized on an industrial scale with copper oxides. Pictures in Figure 1 show the PES/CO fabric before (Figure 1a), during (Figure 1b), and after (Figure 1c) functionalization process on an industrial scale. Figure 1a shows the non-functionalized PES/CO fabric or without copper oxides, which is white. In Figure 1b, it can be seen that after impregnation with copper sulfate and sodium hydroxide, the hue of the non-functionalized fabric changed to a greenish blue. Figure 1c shows that the fabric acquired a beige shade with subsequent heat treatments.

**Figure 2:**
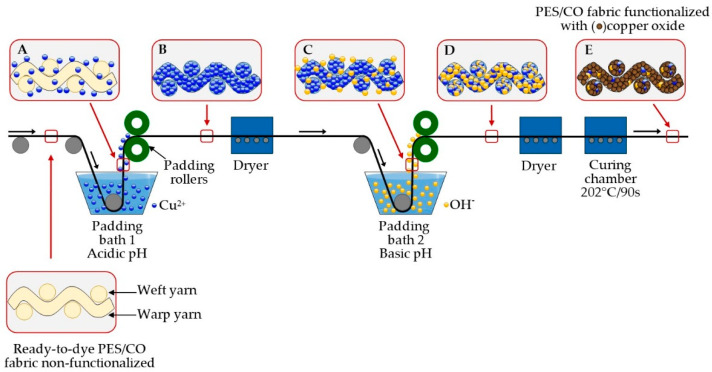
It shows the wet-on-dry process for in situ synthesis of copper oxides on PES/CO fabric. (**A**) Copper ions on the fabric surface of and/or inside its yarns and fibers; (**B**) Absorption of copper ions; (**C**) Hydroxyl ions (OH^−^) on the fabric surface of and/or inside its yarns and fibers; (**D**) Absorption of OH^-^ ions (**E**) Functionalization of the fabric with copper oxides. (Figure is not to scale).

A schematic of the in situ synthesis of copper oxides on PES/CO fabric is illustrated in Figure 2. By using a padder, textile chemical finishes are applied to dyed fabrics after a drying step. Thus, dry fabric passes through the chemical finishing solution, called a wet-on-dry process [40]. The dry ready-to-dye fabric was passed through a padding bath 1 containing Cu^2+^ ions from copper sulfate aqueous solution of acidic pH in the functionalization process. These copper ions were dragged on the fabric surface and/or inside its yarns and fibers (Enlargement A). Due to the pressure exerted by the padding rollers of the padding machine, the adsorbed copper ions were introduced into the interstices of cotton and polyester yarns and fibers (Enlargement B). Then, the dry PES/CO fabric was passed through an extra padding bath 2 containing NaOH solution, and the fabric again dragged ions, in this case, hydroxyls (OH^−^) (Enlargement C). The fabric was pressed between the padding rollers, and the OH^−^ ions entered the interior of the fibers, thus producing a fabric containing Cu^2+^ and OH^−^ ions (Enlargement D). When the temperature reaches 202 °C, and during 90 s, the ions react, giving Cu^1+^ and Cu^2+^ oxides or the mixture of both oxides occurring in the fabric (Enlargement E) by in-situ synthesis method. The synthesized oxides remained on the fabric after neutralization and rinsing with water. Therefore, copper oxides were in situ synthesized on PES/CO fabric on an industrial scale.

**Figure 3:**
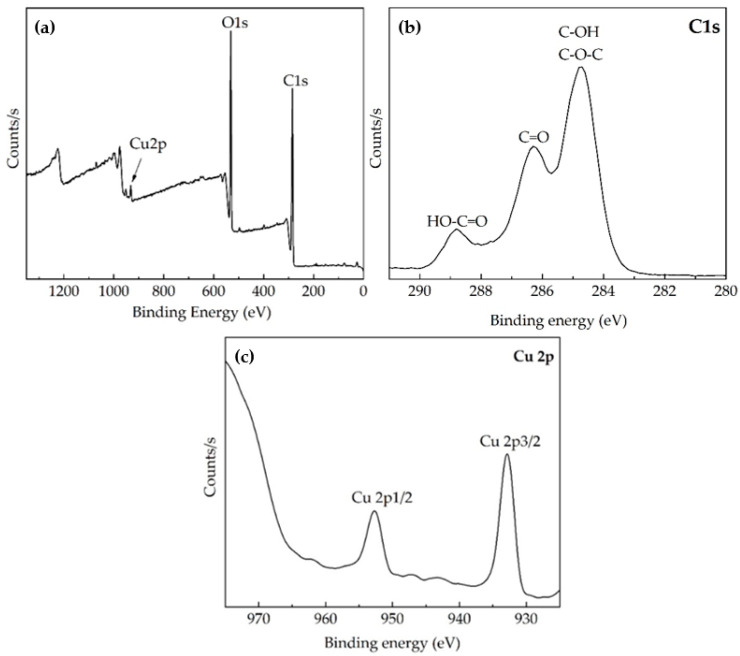
XPS spectra of PES/CO fabric functionalized with copper oxides (**a**); high-resolution for Cs (**b**) and Cu 2p (**c**).

XPS analysis provides information about the surface elemental composition and functional groups. XPS spectra of the PES/CO fabric functionalized with copper oxides are shown in Figure 3.

In Figure 3a, the broad XPS spectrum of the PES/CO fabric shows two peaks with binding energies at 532.5 and 284.7 eV corresponding to oxygen (O1s) and carbon (C1s), respectively, attributed to cotton and polyester fibers [41,42]. The binding energy around 932 eV is assigned to the photoelectron peak of copper (Cu 2p) [43]. According to Figure 3b, the three peaks at high-resolution C1s spectra of functionalized fabric with binding energies at 288.7, 286.3, and 284.7 eV correspond to carbon to carbon or hydrogen in benzene ring (C–C, C–H), methylene carbons singly bound to oxygen (C–O–C), and C double bond O groups [44,45]. Figure 3c displays peaks with binding energies close to 933 eV and 953 eV assigned to Cu 2p3/2 and Cu 2p1/2, respectively, corresponding to copper oxides (Cu_2_O or CuO) [46]. These results indicate the existence of copper oxides on the PES/CO fabric, which could form some type of physical or chemical interaction with the cotton cellulose and polyester. While there are possible bonding interactions between fabric and oxides, it remains to be seen whether bonding occurs at the surface and/or with internal fibers. Copper compounds such as cuprous oxide (Cu_2_O) and cupric oxide (CuO) are red and black, respectively [47]. According to Huang et al. (2020), the contents of Cu^1+^ and Cu^2+^ on the surface of polyester fabrics coated with copper oxides affected their hue [48]. Therefore, the beige shade appearance of the fabric is due to the functionalization on an industrial scale (seen Figure 1c) and may be related to the presence of copper oxide particles on its matrix and/or surface [22,48].

**Figure 4:**
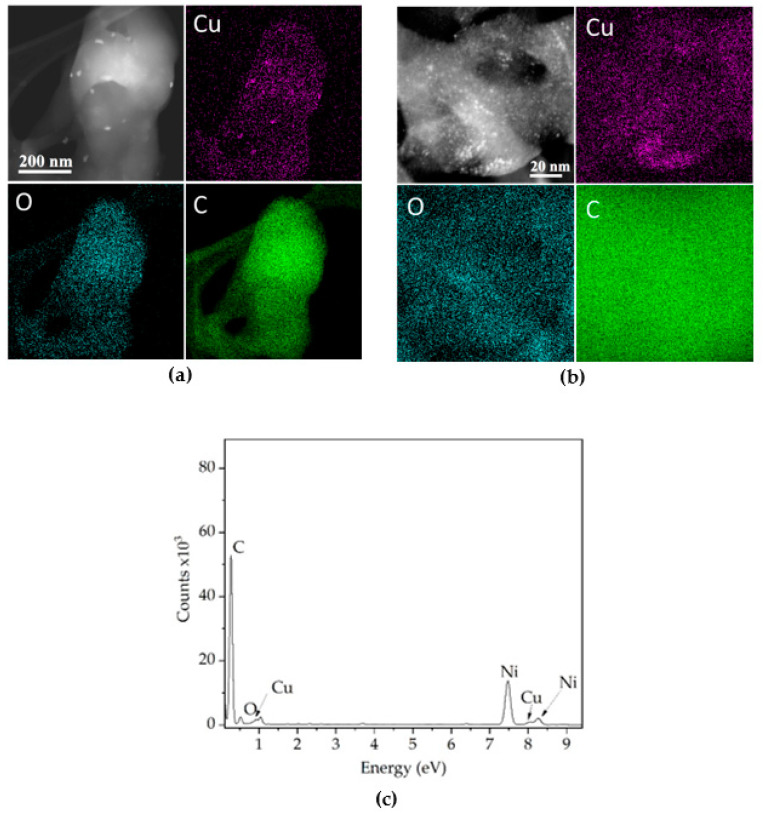
Elemental mapping images according to the copper oxide particle size around (**a**) 20 nm and (**b**) 3 nm (**c**) EDS spectra.

Figure 4a shows the elemental analysis mapping acquired by STEM mode. Copper oxide particles are distributed on the surface of the polyester–cellulose fiber, whose diameter is 400 nm. The size of the particles is around 20 nm. The polyester–cellulose composition is C and O. According to the Z-contrast image, the position of each particle correlates with the Cu element intensity. Figure 4b shows the elemental analysis mapping of tiny particles of copper oxides distributed on the polyester–cellulose fiber; the particle size is around 3 nm. The Cu and O element distribution adequately correlates with the particle’s position at the Z-contrast image. Finally, Figure 4c shows the EDS spectra, and the intensity for each element (Cu and O) can be noticed. The elemental Ni observed in the spectrum was generated from the nickel grid used for STEM.

The results of the color coordinates in the CIELAB space of the non-functionalized and functionalized fabrics were as follows. The non-functionalized fabric had a lightness (L*) value of 92.79, a* and b* color coordinates of −0.31 and 2.08, respectively. These values indicate that the fabric has a yellow shade with high luminosity. After the functionalization process, the L* of the non-functionalized fabric decreased to a value of near 80. Opposite behavior was presented by a* and b* coordinates, where a* and b* increased until 2.11 and 11.43, respectively. These variations show that the fabric presented a darker reddish-brown (beige) shade than the shade of the non-functionalized fabric, which is associated with the copper oxides seen by XPS and STEM.

The quality control results related to the colorfastness of the PES/CO fabric functionalized with copper oxides are shown in Table 1. In colorfastness to laundering, it was observed that there was no transfer of the color of the functionalized fabric towards the acetate, cotton, nylon, polyester, and acrylic fibers. For the wool fiber, appreciable staining was perceived. In the case of the color change evaluation, a grade of 3 indicates that the color of the functionalized fabric had a moderate change. In colorfastness to acid perspiration, the results showed that the color of the functionalized fabric had negligible transfer or staining to all fibers of the multifiber test fabric. For a color change, a grade of 2.3 was observed. This suggests that the color of the functionalized fabric had an appreciable to moderate change. Regarding the colorfastness to crocking, the color transfer of the functionalized PES/CO fabric towards the dry and wet white test was grade 5 and 4, respectively. These values mean that there was no staining towards other fibers. The functionalized fabric had an appreciable color change in colorfastness to light when it was irradiated with light. Therefore, the copper oxides that give color to the fabric after the functionalization process has high resistance to being transferred to adjacent materials when subjected to laundering, perspiration, and crocking. This behavior may be due to forming some type of bond between the copper oxides and the cotton and polyester fibers.

The quality control results related to the physical properties of the fabric with and without functionalization are displayed in Table 2. It can be noticed that the amount of yarn in the weft direction remains almost constant for both samples, whereas in the warp direction, there is an increase in the amount of yarn for the functionalized sample. This increase is associated with a slight shrinkage of the fabric in the warp direction. Likewise, after the functionalization process, the breaking strength of both warp and weft yarns increases between 3 and 6%. This effect may be attributed to higher warp yarn density than weft yarn density. For the case of tearing strength, Mukhopadhyay et al. (2006) found that the tearing of fabrics is very susceptible to process changes during its manufacture. Furthermore, they observed that due to the dying of a fabric composed of the same yarn, its tearing strength fell by approximately 39% and 24% for the warp and weft, respectively. It was attributed to the increased stiffness and decreased extensibility of the yarn and the weakening of the yarn surface parameters such as yarn diameter and contact pressure between warp and weft [49]. Based on this research, it could be assumed that the fall of warp (~24%) and weft (~34%) tearing strength of our fabric functionalized with copper oxides may be related to some change in the yarn properties and fabric surface. In addition, this tearing strength loss in finished fabrics is expected behavior in the fabric manufacturing industry. The quality control results show that the functionalized fabric has good colorfastness, and the copper oxides did not drastically change their physical properties.

Textile’s antiviral and antibacterial properties with and without copper oxides were assessed based on positive quality control results. A quantitative method was applied to determine the antiviral activity of fabrics according to the ISO 18184:2019 [38]. SARS-CoV-2_COV2019 ITALY/INMI1 and Human Corona Virus (229E) were the strains used. The test consisted of putting fabrics with and without functionalization in contact with a virus dispersion. The difference between the initial viral titer resulting from the first instantaneous recovery after inoculation on non-functionalized fabric (time zero) and the residual viral titers of functionalized fabric after 30 and 60 min of contact was used to assess antiviral activity. The viral titer was determined by the TCID_50_ method.

**Figure 5:**
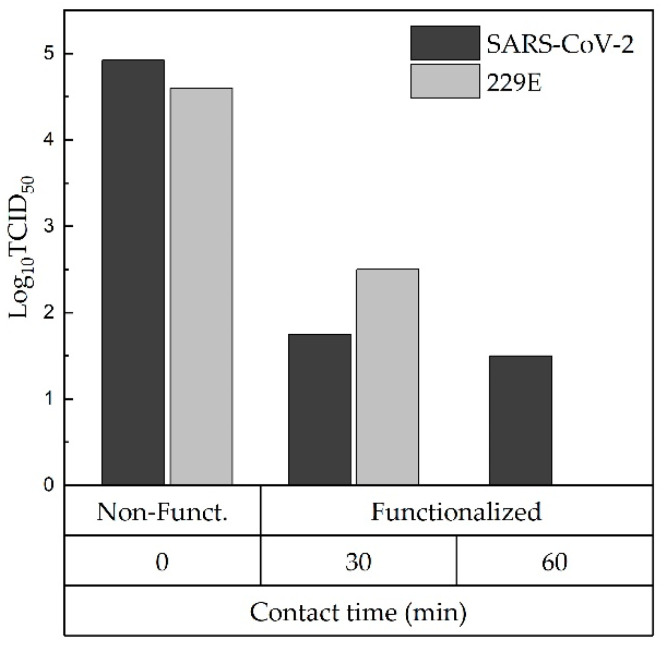
Achievement of TCID_50_-titres as a function of contact time of non-functionalized and functionalized fabrics with the SARS-CoV-2 and 229E viruses.

In Figure 5, the antiviral activity assessment revealed a decrease in viral titer of the functionalized fabric concerning the non-functionalized fabric of 3.17 and 2.5 log_10_ TCID_50_ for SARS-CoV-2 and 229E, respectively, in 30 min exposure. After 60 min of exposure, the viral titer continued to decrease until reaching a value of 1.50 log_10_ TCID_50_ for the case of SARS-CoV-2. With these results and based on international standards ISO 18184:2019, the virucidal activity of fabric functionalized with copper oxides against SARS-CoV-2 is considered excellent, with an inactivation efficiency of 99.93% and 99.96% in 30 and 60 min exposure, respectively; while against the 229E strain, virucidal activity is considered as good. A recent study developed by Camero et al. (2021) evaluated the virucidal activity of a cotton fabric treated with copper derivative against feline coronavirus (FCoV) as a surrogate of SARS-CoV-2; after 2 h of incubation, a decrease in viral titer of 2.58 log_10_ TCID_50_/50 μL was observed in treated fabric, whose antiviral efficacy was considered good [50]. Similarly, Hewawaduge et al. (2021) demonstrated a three-layer mask made of polyester, nylon, and spandex fibers incorporating copper sulfide (CuS) inactivated SARS-CoV-2 after 30 min exposure [51]. Other studies showed that when coating solid surfaces with cuprous oxide (Cu_2_O) and cupric oxide (CuO), SARS-CoV-2 was 99.9% inactivated within 1 h exposure [52,53]. Therefore, according to the findings in this report and the literature, copper’s efficacy is demonstrated, either as Cu^1+^ or Cu^2+^, to deactivate SARS-CoV-2 and other coronaviruses [51,52,53]. The antiviral properties of copper are due to the release of Cu ion that can damage viral lipid membranes and nucleic acids, leading to inactivation of the virus [51,54], and also due to the production of reactive oxygen species (ROS), which are capable of damaging viral proteins and lipids [55,56,57].

Apart from the problems caused by SARS-CoV-2 in health clinics such as shortage of medical equipment, PPE, and medicine; a collapse in service provision; mental problems in health workers; and infections produced during hospitalization (HAIs) by human pathogens such as *Escherichia coli*, *Staphylococcus aureus*, *Pseudomonas aeruginosa*, *Klebsiella pneumoniae*, and others, should be considered. It is well known that HAIs are often caused by surgical wounds, respiratory infections, cross-infection, or immune response infections [58].

**Figure 6:**
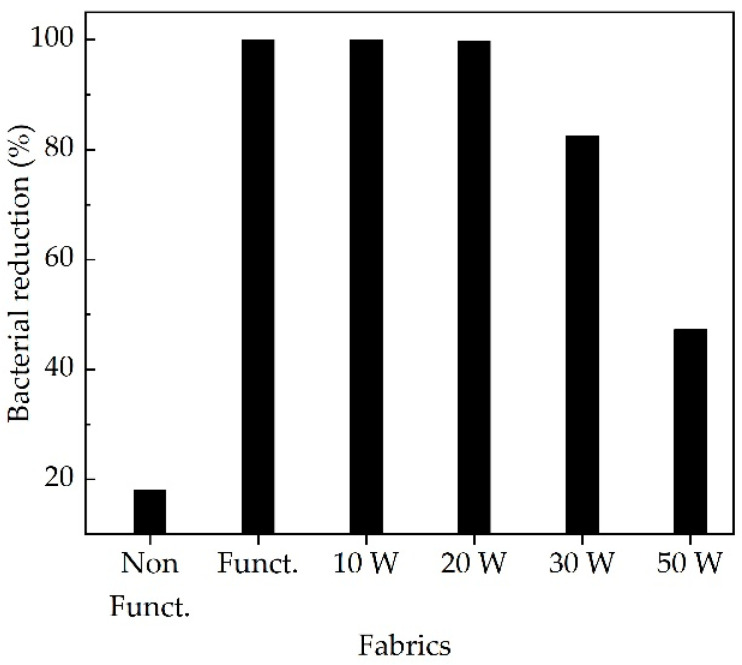
Results of a percentage of bacterial reduction for *Escherichia coli* (ATCC 25922), according to ASTM E2149 standard.

The results of the percentage reduction in the bacterial cell population of the PES/CO fabrics, before and after washing cycles, evaluated according to the technical standard ASTM E2149—2013, are found in Figure 6. The *Escherichia coli* (ATCC 25922) strain was found to be inhibited by the functionalized fabric with copper oxides with a reduction percentage of 99.9%. This percentage corresponds to a copper quantity of 728 ppm. After 10 and 20 washes, copper oxides were reduced to 497 and 300 ppm, respectively. Despite this post-washing reduction in the copper particles, functionalized fabric continued to reduce bacterial growth by more than 99.7%. As the number of washes increased, the reduction percentage fluctuated wildly. The functionalized fabric had an 82.5% reduction after 30 washes, while lower rates were detected after 50 washes, with a value of 47.3%. This loss of antimicrobial activity is probably related to the decrease in copper oxide quantity on the fabric surface, produced by constant friction between steel spheres and fabric during its washing time.

Therefore, from the results of STEM, XPS, and the resistance of the functionalized fabric to maintain its antimicrobial properties against domestic washing cycles, it is possible to infer the existence of some type of physical or chemical interaction between copper oxides and cellulose fiber and polyester fiber.

**Figure 7:**
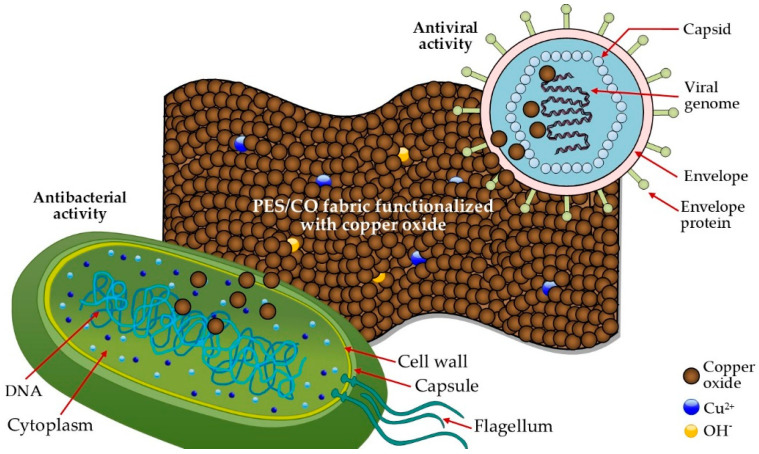
Scheme of the antibacterial and antiviral activity of the PES/CO fabric functionalized with copper oxides.

The main mechanisms of the antimicrobial activity of the copper oxides are not yet fully understood. Copper oxide’s antibacterial properties are attributable to various mechanisms such as the release of copper ions, the direct contact of copper oxide with bacteria, and the ROS, which, when in connection with the components of the bacterial cell envelope (cell wall and cytoplasmic membrane), cause their death [19,59,60]. Copper’s antiviral activity may be associated with the release of copper ions and ROS generation [61,62]. Copper can cause irreversible damage to the viral membrane, disintegrate the envelopes and destroy the genomic material of viruses; in addition, it can inhibit virus replication [62,63]. Figure 7 shows a schematic representation of the antimicrobial activity of the PES/CO fabric functionalized with copper oxides.

The cytotoxicological analysis of textiles functionalized with copper oxides is still under investigation. In preliminary qualitative tests carried out by this research group, the fabric functionalized with copper oxides was not toxic, but these results should be repeated and studied in more depth.

The copper oxide particles present in the functionalized PES/CO fiber were forcibly separated by ultrasound to visualize by electron microscope technique (STEM mode). It is common knowledge that when we wear textile garments, there is friction between the human skin and the fabric or between fabric and fabric. Then, a way to usually remove the copper oxides from the fabric would be by crocking, but it does not have the same intensity as ultrasound. Consequently, the copper oxides are not easily detached from the fabric and would not represent a risk to the health of the users. The results of colorfastness have corroborated this statement to crocking discussed above (Table 1) since this colorfastness was determined if the copper oxides of the functionalized PES/CO fabric were transferred to other surfaces by crocking.

## 4. Conclusions

The impregnation dyeing method was used to functionalize 265 m of PES/CO fabric with copper oxides on an industrial scale. Copper oxides were in-situ synthesized on the fabric using a technical grade of copper salt precursor. The copper oxide particles are distributed on the polyester–cellulose fiber with a size of around 3 nm and 20 nm. XPS confirmed that there are copper oxides on the surface fabric after the functionalization process, which are responsible for the beige shade of the fabric and have good colorfastness. In addition, the physical properties did not drastically change. It was also demonstrated that the fabric functionalized with the copper oxides inactivated the SARS-CoV-2 and 229E viruses. According to the ISO 18184 standard, the viral activity of the fabric against these two strains was considered excellent and reasonable. It should also be mentioned that the inactivation efficiency against SARS-CoV-2 was 99.93% and 99.96% in 30 and 60 min exposure, respectively. However, the fabric with copper oxides still reduced more than 99% of the growth of *Escherichia coli* bacteria before and after 10 and 20 washes. Therefore, reusable face masks made from fabrics with antiviral and antibacterial properties functionalized with copper oxides would prevent SARS-CoV-2 and other pathogens and reduce environmental contamination.

## Figures and Tables

**Table 1:** Colorfastness of functionalized fabric evaluated with greyscale for color change and staining.

Evaluation			Colorfastness			
			Laundering	Perspiration	Crocking	Light
Staining	Multifiber test fabrics	Acetate	5	4.5	---	---
		Cotton	4.5	4.5	---	---
		Nylon	5	4.5	---	---
		Polyester	5	4.5	---	---
		Acrylic	4.5	4.5	---	---
		Wool	2.5	4.5	---	---
	White test	Dry	---	---	5	---
		Wet	---	---	4	---
Color change			3	2.3	---	2

**Table 2:** Physical properties of PES/CO fabric.

Fabric	Yarn Density		Breaking Strength (N)		Tearing Strength (N)	
	Ends/In	Picks/In	Warp	Weft	Warp	Weft
Non-functionalized	147	82	769	368	22	12.5
Functionalized	155	81	792	393	17	8.2

## Data Availability

Not applicable.
